# Differences in In Vitro Bacterial Adherence between Ti6Al4V and CoCrMo Alloys

**DOI:** 10.3390/ma16041505

**Published:** 2023-02-10

**Authors:** Marta Martín-García, John Jairo Aguilera-Correa, María Ángeles Arenas, Ignacio M. García-Diego, Ana Conde, Juan José de Damborenea, Jaime Esteban

**Affiliations:** 1 Department of Clinical Microbiology, University Hospital Fundación Jiménez Díaz, IIS-FJD, 28040 Madrid, Spain; 2 CIBERINFEC—CIBER de Enfermedades Infecciosas, Instituto de Salud Carlos III, 28029 Madrid, Spain; 3 Department of Surface Engineering Corrosion and Durability, National Centre for Metallurgical Research (CENIM-CSIC), 28040 Madrid, Spain

**Keywords:** bacteria, adherence, Ti6Al4V, CoCrMo, alloy, prosthesis

## Abstract

Prosthetic joint infection is an uncommon entity, but it supposes high costs, both from the economic side to the health systems and from the emotional side of the patient. The evaluation of the bacterial adherence to different materials frequently involved in joint prostheses allows us to better understand the mechanisms underlying this and provide information for the future development of prevention strategies. This study evaluated the bacterial adherence of four different species (*Staphylococcus aureus*, *Staphylococcus epidermidis*, *Escherichia coli* and *Pseudomonas aeruginosa*) on Ti6Al4V and CoCrMo. The topography, surface contact angles, and linear average roughness were measured in the samples from both alloys. The interaction with the surface of both alloys was significantly different, with the CoCrMo showing an aggregating effect on all the species, with additional anti-adherent activity in the case of *Pseudomonas aeruginosa*. The viability also changes, with a significant decrease (*p* < 0.05) in the CoCrMo alloy. In the case of *S. epidermidis,* the viability in the supernatant from the samples was different, too, with a decrease in the colony-forming units in the Ti6Al4V, which could be related to cation release from the surface. Beyond adhesion is a multifactorial and complex process, and considering that topography and wettability were similar, the chemical composition could play a main role in the different properties observed.

## 1. Introduction

The use of orthopaedic prostheses has been a significant advance in medicine, allowing improved quality of life for millions of patients. However, this advance is not exempt from inconveniences; among them, infection is one of the most important because of the increase in morbidity, mortality, and healthcare-associated costs [1,2]. Nowadays, the 5-year survivorship after hip and knee PJI is less than that found in breast and prostate cancer [3]. Despite the incidence being low—between 1–3% in most of the knee and hip joint replacement series [4]—the absolute number of patients with prosthetic joint infection (PJI) is increasing, as the number of patients that undergo arthroplasty procedures is also expected to increase. This fact must also consider the importance of this disease from several points of view. Regarding morbimortality, PJI increases hospitalisation times, can lead to secondary infections, and even can affect the functionality of the affected joint [4,5]. In some cases, amputation may be needed if a cure for the infection (or at least its control) is to be achieved. Moreover, mortality can also be high among these patients [6]. Another critical point of view is the economic burden of this disease. The cost of one PJI can be as high as US$100,000 or even more [5]. This amount can be even higher if other aspects of treatment (such as new surgeries, antibiotic therapies, new admissions, and others) are considered. All these factors make PJI a fundamental problem in modern medicine.

The most common microbial aetiology in PJI is staphylococci, including *Staphylococcus aureus* and different species of coagulase-negative staphylococci, *Staphylococcus epidermidis* being the most commonly isolated among them [7]. However, almost all microorganisms can be considered a cause of PJI if they reach the implant [8]. Moreover, there are cases in which the infection is caused by many different species of bacteria (polymicrobial infections). On the other hand, sometimes, no microorganisms can be detected in the culture (culture-negative infections). The knowledge of aetiology has improved with the use of modern molecular methods that allow the detection of non-culturable microorganisms, especially with the help of new molecular tools based on metagenomics and next-generation sequencing [9]. However, despite all the advances in microbiological diagnosis, most infections are still caused by a relatively small group of microorganisms.

Prosthesis components are usually made of an ultra-high molecular weight polyethylene part together with other parts manufactured from a combination of metal alloys with high corrosion resistance, mechanical strength, and hardness, being also biocompatible and improving osseointegration. These alloys are usually AISI 316L, Ti6Al4V, and for some specific parts requiring good wear properties, CoCrMo. Ti6Al4V presents an excellent strength to modulus ratio to avoid stress shielding, but its wear resistance is poor compared to that of CoCrMo. However, it has the advantage of better osteointegration, and because of this reason, it is usually found in the fixed part of the prosthesis. Therefore, CoCrMo is still used for specific parts of the prosthesis, such as the femoral knee component and the femoral head of hip joints, where better wear resistance is required, but no important osteointegration is necessary.

Biofilm is a community of bacteria aggregated in a self-produced polymeric matrix composed of exopolysaccharides, proteins, and extracellular DNA with a complex behaviour that provides advantages facing adverse conditions such as the defence against the immune system and the resistance to antibiotics [10]. Biofilm formation involves different stages: adhesion, microcolony formation by aggregation and the polymeric extracellular matrix production, and macrocolony formation due to maturation that can lead to the release of planktonic bacteria [11]. The first step in biofilm development is always the adherence of the microorganisms to a surface. This phenomenon is a complex process that usually involves a two-step process [12]. In the first one (attachment), there are physical forces (such as Van der Waals forces, Brownian movement, electrostatic charges, and others). This step is followed by a chemical phase (adhesion) where covalent links are established between bacteria and the surface. This phase is influenced by the presence of different host molecules on the implant surface. Both steps are dynamic processes that cannot be divided strictly [12].

We have the most important chance to prevent biofilm development during the adhesion stage because the bacteria in the planktonic state are easier to eradicate. Therefore, many surface modifications have been designed to decrease or even avoid the adherence of microorganisms to the implant surface. Unfortunately, data about in vitro adherence of several species of bacteria to Ti6Al4V and CoCrMo are scarce [13,14] and mainly aimed to study Gram-positive organisms, especially *Staphylococcus aureus*. However, recent data suggest that the role of Gram-negative organisms in PJI is increasing [8], and the problem of antimicrobial resistance among these organisms is a matter of particular concern [15,16]. In this context, it is extremely important to avoid bacterial adherence to the different components of the implants because the management of infections caused by these microorganisms is extremely difficult and, in some cases, almost impossible.

This work evaluates the influence of the substrate on the adherence of Gram-positive and Gram-negative bacteria on both Ti6Al4V and CoCrMo alloys to gain some knowledge of the differences between these groups of bacteria and their interaction with these two metal alloys of different chemical compositions widely used in arthroplasties.

## 2. Material and Methods

An 18 mm diameter rod of the Ti6Al4V alloy of ELI grade, manufactured according to the standard ASTM F136-02, was supplied by Surgical Co., SAU (Valencia, Spain). The rod was cut into 2 mm thick disc specimens and ground through successive grades of SiC paper to 2000 grade. Subsequently, a chemical-mechanical polishing was carried out in a commercial colloidal silica suspension (OP-S Suspension. 0.25 µm from Struers, Copenhagen, Denmark) with hydrogen peroxide with a volume ratio of 9:1 to achieve a smooth finish with a final controlled roughness.

A 19 mm in diameter rod of the CoCrMo alloy was supplied by Carpenter Technology Corp. (Philadelphia, PA, USA), melted and complied with ASTM F1537-11. The rod was cut into discs of 2 mm thickness. One face of each specimen was ground on silicon carbide papers and polished to a 3 µm diamond finish. Table 1 summarises the chemical composition of the CoCrMo alloy given by the supplier.

After mechanical polishing, the specimens of both alloys were repeatedly rinsed with distilled water, rinsed in ethanol, and dried in a cool air flow.

SEM examination of both alloys was carried out using a Hitachi S-4800 from Hitachi High Technology Science Corporation (Tokyo, Japan) equipped with energy-dispersive X-ray (EDX) instruments to characterise the surface and morphological features of the alloys. Linear average roughness, Ra, values of both alloys were obtained using an optical imaging profilometer Plμ 2300 (Sensofar) (Terrassa, Spain) operated at 20×. The R_a_ values are an average of sixteen measurements. Surface contact angles from the alloys were measured with distilled water using Theta Attension optical tensiometer (KSV Instruments, Monroe, CT, USA) with an automatic liquid dispenser and monochromatic cold light source, operated in trigger mode with 50 video frames recorded at a 112 ms interval. Contact angles were calculated using the Young-Laplace drop profile fitting method. Each contact angle value is cited as an average of three measurements performed at three different locations on the specimen surface. An average of 40 frames has been used to calculate the contact angle for each drop.

*S. aureus* ATCC 29213, *S. epidermidis* ATCC 35984, *P. aeruginosa* ATCC 27853, and *E. coli* ATCC 25922 collection strains were used in the experiments. They were kept frozen until the experiments were performed. The bacterial adherence study was performed according to the methodology previously described by Aguilera-Correa et al. [17]. Briefly, samples were washed and vortexed for 30 s at 3000 rpm in pure distilled water (B. Braun, Melsugen, Germany). The strains were cultured in tryptic soy broth (BioMerieux, Marcy-l’Etoile, France) at 37 °C for 24 h, then centrifuged at 3500 rpm at 22 °C for 10 min. The supernatant was removed, and the pellet was washed three times with sterile 0.9% NaCl saline solution (SS) (B. Braun, Melsungen, Germany) and then resuspended and diluted in SS to 10^8^ CFU/mL bacterial solution. Both types of alloy coupons were incubated in 5 mL of this solution in a sterile nontreated six-well plate (Thermo Fisher Scientific, Waltham, MA, USA) at 37 °C for 90 min to allow bacterial adhesion. After incubation, discs were washed three times with SS. Samples were stained with LIVE/DEAD BacLight Stain (Thermo Fisher Scientific, MA, USA) and the images obtained were analysed using ImageJ software (National Institutes of Health, Bethesda, MD, USA).

Bacterial counting and percentage of the covered surface were calculated with the ImageJ software (National Institute of Health, Bethesda, USA), using ten images for each coupon, taken from the same places with 40× magnification in a fluorescence microscope (DM 2000 Fluorescence microscope Leica, Germany). Viability was calculated by comparing the number of green vs. red stained cells in ten images for each coupon. The viability of planktonic bacteria after incubation was also evaluated by counting the colonies recovered from the supernatant by the drop-plate technique. The experiments were performed in triplicate.

The statistical analysis was performed using GraphPad Prism 8. Data were analysed by the nonparametric unilateral Wilcoxon test with a level of statistical significance of *p* < 0.05. The values are cited as medians and interquartile ranges.

## 3. Results

**Figure 1:**
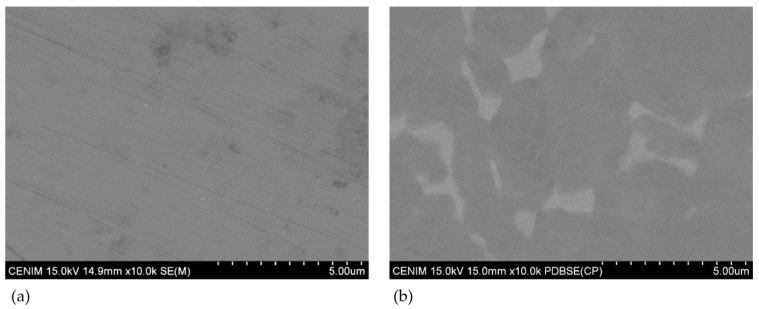
SEM appearance of (**a**) mechanical CoCrMo alloy polishing and (**b**) chemical-mechanical Ti6Al4V alloy polishing after surface preparation.

Figure 1 shows the surface appearance of both alloys after surface preparation (mechanical polishing for the CoCrMo alloy and chemical polishing for the Ti6Al4V alloy). No characteristic features (only some grinding lines) are observed on the surface of the CoCrMo alloy (Figure 1a), while the α + β phase morphology of the Ti6Al4V alloy is seen in Figure 1b. This morphology comprises β phases, 2–3 µm long, of irregular shapes in an α matrix. Average roughness, Ra, values were very similar for both alloys. Thus, Ra was 16.1 ± 2.7 nm for the CoCrMo alloy and 13.7 ± 1.9 nm for the Ti6Al4V alloy.

Both alloys showed similar values for the contact angle measured for distilled water, 54.6 ± 0.6° and 52.10 ± 8.2° for CoCrMo and Ti6Al4V alloys, respectively, pointing out that both metals have a hydrophilic nature.

**Figure 2:**
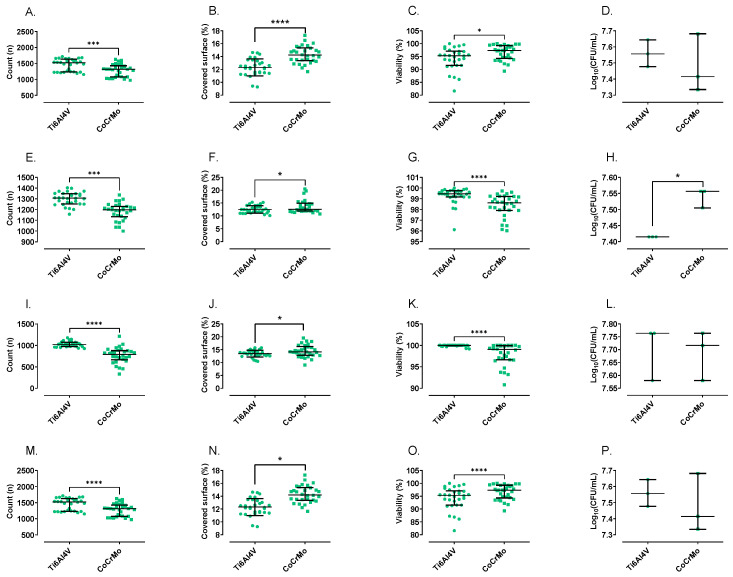
Counts (**A**,**E**,**I**,**M**), covered surface (**B**,**F**,**J**,**N**), adhered bacteria viability (**C**,**G**,**K**,**O**), and supernatant bacterial colony-forming units per millilitre (**D**,**H**,**L**,**P**) of *S. aureus* (**A**–**D**), *S. epidermidis* (**E**–**H**), *E. coli* (**I**–**L**), and *P. aeruginosa* (**M**–**P**). * *p* < 0.05; *** *p* < 0.001; **** *p* < 0.0001.

**Figure 3:**
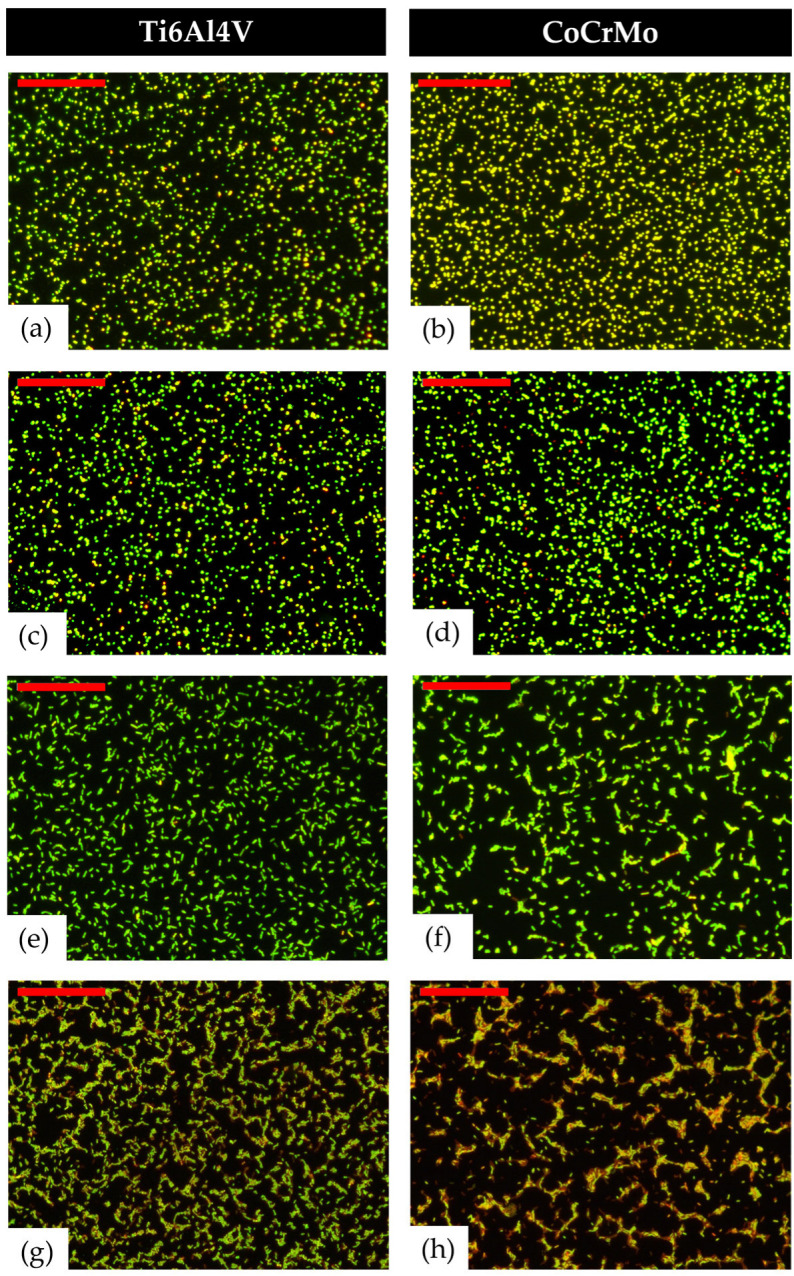
Images of *S. aureus* (**a**,**b**), *S. epidermidis* (**c**,**d**), *E. coli* (**e**,**f**), and *P. aeruginosa* (**g**,**h**) adhered on Ti6Al4V alloy (**a**,**c**,**e**,**g**) and CoCrMo alloy (**b**,**d**,**f**,**h**). DM 2000 Fluorescence microscope. The red bar represents 50 µm.

For all tested species, bacterial adherence was significantly different between the Ti6Al4V and CoCrMo alloys (Figure 2 and Figure 3). In the case of the bacterial count, all bacterial species showed a lower count of organisms on the CoCrMo alloy (*p* < 0.0001), with a significant reduction of 3.5%, 8.3%, 23% and 55.6% for *S. aureus*, *S. epidermidis*, *E. coli* and *P. aeruginosa*, respectively, the highest difference being the one found between both alloys and *P. aeruginosa*. The surface area covered by bacterial cells was 13.7%, 1.8% and 5.1% higher on the CoCrMo alloy than on the Ti6Al4V alloy for *S. aureus*, *S. epidermidis* and *E. coli,* respectively. The highest covered area was observed for *S. aureus* (*p* < 0.0001). Meanwhile, for *P. aeruginosa* (*p* < 0.05), the covered area was 15.6% lower than on Ti6Al4V. Beyond the differences among species observed in the covered area and count percentages, it is important to highlight that there were statistically significant differences between both alloys (*p* < 0.05) in all cases. Regarding viability using the BacLight LIVE/DEAD stain, the percentage of viable organisms on the surface was significantly lower on the CoCrMo alloy for all species, with a reduction of 2.3%, 0.8%, 0.95%, and 10.6% for *S. aureus*, *S. epidermidis*, *E. coli* and *P. aeruginosa*. Regarding the concentration of planktonic bacteria, there were no significant differences between both alloys for *S. aureus*, *E. coli* and *P. aeruginosa.* Conversely, *S. epidermidis* showed higher colony-forming units per millilitre (CFU/mL) for the CoCrMo alloy compared to the Ti6Al4V alloy, showing 27.7% less CFU/mL (*p* < 0.05).

## 4. Discussion

Bacterial adherence to different surfaces is considered the first step in the pathogenic process of all infections. In PJI, this adherence is affected by the “race for the surface” theory, and only when bacteria first adhere to the implant surface during surgery does biofilm develop, and infection arises [18,19].

The implant of a foreign body alters the granulocyte activity due to the release of defensins that decrease phagocytic activity [20], making the surface characteristics of the implant even more relevant.

Because prostheses are usually made of different alloys in their parts according to the biomechanical and biocompatibility needs, the infection could appear more easily in those showing lower antibacterial properties. Both Ti6Al4V and CoCrMo alloys are commonly used in the metallic components of articular prostheses currently employed, so differences in initial bacterial adherence between alloys might lead to different subsequent infection susceptibility scenarios. This could be due to several reasons and may be different depending on the bacterial species. In addition to the type of bacteria, the physicochemical properties of the alloys might influence bacterial adhesion.

For this study, we chose bacteria that are commonly isolated from PJI. Coagulase-negative staphylococci are the most frequently found bacteria, followed by S. aureus [7]. In addition, Gram-negative bacilli are gaining relevance in recent years, being especially relevant in early post-interventional PJI [8]. Among the latter, *E. coli* is the most isolated, but species such as *P. aeruginosa* are very relevant as they represent a challenge from the treatment point of view. In this sense, it is important to note that the distribution of the different species that cause PJI is different regarding the type of infection. In the article of Benito et al. [7], acute infections are more frequently caused by *S. aureus* or Gram-negative bacilli, being *S. epidermidis* and other less pathogenic organisms more frequently isolated from chronic PJI. Moreover, polymicrobial infections are usually acute PJI, while this type of infection is uncommon among chronic patients [5,7].

Surface factors related to bacterial adherence include chemical structure, surface roughness, hydrophilicity, Z-potential and surface-free energy, all of which have been found to influence bacterial adhesion [21]. In our study, the wettability, surface roughness, and topography of the CoCrMo alloy and the Ti6Al4V alloy are very similar. Therefore, the chemical composition of the alloys seems to play a key role in bacterial adherence. Different studies have tried to evaluate the presence of different microorganisms in the different components of the implant. For example, Lass et al. [22] observed some differences between the different parts of a hip prosthesis using sonication, the polyethylene liners being the components with higher bacterial loads of *S. epidermis*, followed by the femoral head made of Ti and the stem manufactured with the Ti6Al7Nb alloy. Conversely, Gomez-Barrena et al. [23], also using sonication as the tool to perform colony-forming unit counts, showed a high variability of the results showing no clear differences between components after an adjustment of the bacterial counts according to the component surface was performed. In the latter study, the number of colonies was adjusted to the implant surface by using a specifically designed program that allows the calculation of the measure of the implant surface. However, the fact that these parts usually include both types of alloys (with different roughness) made it difficult to establish the actual role of chemical composition in the development of infection.

Another approach to evaluate bacterial adherence to the different parts of the implant was developed by the Ohio State University group. In a first in vitro study, Moore et al. inoculated a strain of *S. aureus* on the surface of different orthopaedic implants, incubated them, and measured the number of bacteria present in the different parts of the implant. The results showed that rough surfaces have higher colony counts than smooth surfaces. Of particular interest, the edges showed higher colonisation than other parts of the surfaces, as well as other specific parts of the implant (ridges, screw holes) [24]. More recently, Brooks et al. studied clinical implants from patients with and without a diagnosis of PJI using a thin coat of agar on the entire surface. Using this approach, the authors found an irregular distribution of biofilms on all of the implants’ surfaces, except in the ridges and edges, which appeared consistently positive. Moreover, non-articulating surfaces also showed consistent bacterial growth [25]. These differences can be due to the different shapes of these parts, but the different chemical compositions can also influence the differences in biofilm distribution. A limitation of both studies is the relatively low number of studied implants. In the clinical one, only nine patients had the MSIS criteria for infection, and in all but two cases, the cause of the infection was Gram-positive microorganisms. Only one case had a polymicrobial infection that included Gram-negative bacilli. One of the cases was caused by *Candida albicans*, an extremely uncommon cause of PJI in all series.

Bacterial adherence to metallic surfaces is mediated by nonspecific forces (Lifshitz-van der Waals, Lewis acid–base, and electrostatic forces) since these bacteria can behave like colloidal microparticles [11,13]. Nonetheless, even complex colloidal adherence models, such as the extended Derjaguin-Landau-Verwey-Overbeek theory, are not able to predict the behaviour of viable bacteria [26]. This could be explained by the fact that viable bacteria are able to interact with the abiotic surfaces and facilitate their own adherence. Our results confirm the hypothesis that there are differences in the adherence parameters between both alloys. Considering the decrease in the count with the increase in the area covered, the CoCrMo alloy favours the appearance of aggregates in all tested species except for *P. aeruginosa* (Figure 3). On this one, *P. aeruginosa* showed a lower adherence, with a decreased percentage of covered surface and a lower number of bacterial counts on the CoCrMo alloy than the Ti6Al4V alloy. In vitro studies using the same alloys as our study showed similar results to previous ones. The study of Watanabe et al., using *S. aureus* and *Cutibacterium acnes*, showed that bacteria recovered from the CoCrMo alloy gave lower counts than those obtained from the Ti6Al4V alloy in both species [13]. However, this study focussed on biofilm development and not bacterial adherence to the tested materials, so the results might be affected by the biological process of biofilm development. Different studies observed that *S. aureus*, *S. epidermidis*, *P. aeruginosa* and *C. acnes* showed an increased adherence to the CoCrMo alloy compared to the Ti6Al4V alloy [15]. If the literature is scarce and controversial regarding bacterial adherence of Gram-positive species, specifically regarding *S. epidermidis*, which is the main bacteria responsible for PJI, it is even lower about Gram-negative bacilli.

It seems that final adherence is multifactorial, and several mechanisms underlie these differences, including idiosyncratic characteristics of bacteria and physic-chemical properties of the biomaterial such as surface roughness, charge, and wettability [27,28]. These differences might have some relevance during the subsequent biofilm development on different surfaces, although this aspect needs further studies related to osseointegration, which also influences susceptibility to develop an infection. In some works comparing in vivo implants of different materials in rabbit models, the CoCrMo alloy was found to be easier to infect than Ti6Al4V [29]. This could be due to the fact that the latter is more biocompatible than CoCrMo, which from the chemical composition side is closely related to the metal ion release caused for the in vivo corrosion [30]. Another theory proposed by the authors is related to the “race for the surface” theory: because Ti6Al4V is more biocompatible, eukaryotic cells can colonise its surface more easily than CoCrMo [18,19]. However, because this is an in vivo model, other variables can influence these results. In the final analysis, the roughness of the material is the most important property of the material that influences the development of infection. Nevertheless, it seems reasonable that higher biocompatibility allows the growth of bone cells on its surface more quickly, protecting the surface from colonisation by bacterial pathogens. CoCrMo biocompatibility and osseointegration can be improved using different strategies that modify the surface [31,32]. This metal ion release could affect microbial metabolism and play an important role in the differences observed in adherence [33].

Moreover, *S. epidermidis* concentration in the supernatant from the CoCrMo alloy samples was slightly but significantly higher than that from the Ti6Al4V alloy. While most of the species showed a decrease in CFU/mL in the presence of the CoCrMo alloy, *S. epidermidis* reveals an increase in viability on CoCrMo compared to Ti6Al4V. This indicates that bacteria from the supernatant can be less aggregated in the presence of this alloy compared to its counterpart in Ti6Al4V. This lower aggregation may also be based on the cation release, which requires further studies to understand the mechanism underlying this effect.

## 5. Conclusions

We have shown differences in the adherence of different Gram-positive and Gram-negative bacteria species between CoCrMo and Ti6Al4V alloys, two of the alloys commonly used in the manufacturing of joint prostheses. These differences can be due to the chemical composition of the material, the differences in bacterial characteristics, or, more probably, both of them. Further studies are necessary to fully understand the mechanisms underlying these differences to beuseful for developing strategies to minimise the adherence on these alloys, which might decrease the number of infected prostheses in the near future.

## Figures and Tables

**Table 1:** Chemical composition (in wt.%) of CoCrMo and Ti6Al4V alloys.

CoCrMo Alloy		Ti6Al4V ASTMF1360	
**Element**	% (in wt.)	**Element**	% (in wt.)
Co	64.67	Ti	Bal.
Cr	27.76	Al	5.50–6.50
Mo	5.56	V	3.50–4.50
Mn	0.79	Fe	max. 0.25
Fe	0.41	O	max. 0.13
C	0.04	C	max. 0.08
N	0.165		
Si	0.62		
S	0.001		
Ni	0.12		

## Data Availability

Not applicable.
